# Metabolic Status and Atrioventricular Block Risk: The Role of Physical Activity

**DOI:** 10.31083/RCM37291

**Published:** 2025-05-20

**Authors:** Ho-Gi Chung, Pil-Sung Yang, Eunsun Jang, Daeun Joung, Daehoon Kim, Hee Tae Yu, Tae-Hoon Kim, Jae-Sun Uhm, Jung-Hoon Sung, Hui-Nam Pak, Moon-Hyoung Lee, Boyoung Joung

**Affiliations:** ^1^Division of Cardiology, Department of Internal Medicine, Severance Cardiovascular Hospital, Yonsei University College of Medicine, 03722 Seoul, Republic of Korea; ^2^Department of Cardiology, CHA Bundang Medical Center, CHA University, 13488 Seongnam, Republic of Korea; ^3^Department of Computer Science and Engineering, Ohio State University, Columbus, OH 43210, USA

**Keywords:** accelerometer, atrioventricular block, metabolic status, physical activity

## Abstract

**Background:**

The relationship between metabolic status as a possible risk factor and predictor of response to moderate-to-vigorous physical activity (MVPA) in atrioventricular block (AVB) remains unclear.

**Methods:**

A total of 82,365 UK Biobank participants without a history of AVB or pacemaker implantation, and who were involved in accelerometer work-up, were chosen for the study population. Metabolic status was classified into two categories, healthy and unhealthy, using modified criteria for metabolic syndrome from the International Diabetes Federation. We used the multivariable Cox proportional model to assess the associations between metabolic status and primary outcome (composite of second-degree AVB or third-degree AVB) or secondary outcomes (each component in the primary outcome and AVB-related pacemaker implantation). The relationship between MVPA min/week and the primary outcome in each metabolic status category was assessed using restricted cubic splines.

**Results:**

Of the 82,365 participants, the mean age was 62.3 years, and 44.1% were men. In total, 299 primary outcome events occurred during the 6.1-year follow-up. Compared to metabolically healthy participants, metabolically unhealthy participants had a 58% higher risk of the primary outcome (hazard ratio (HR): 1.58, 95% confidence interval (CI): 1.25–2.00; *p* < 0.001). This pattern was consistent for second-degree AVB (HR: 1.59, 95% CI: 1.12–2.27; *p* = 0.010), third-degree AVB (HR: 1.50, 95% CI: 1.12–2.03; *p* = 0.008), and AVB-related pacemaker implantation (HR: 2.25, 95% CI: 1.44–3.52; *p* < 0.001). Increased MVPA provided statistically significant protection against the primary outcome only in metabolically unhealthy participants, with a threshold of 830 min/week.

**Conclusions:**

Generally, in the middle-aged population, metabolically unhealthy participants had a statistically significantly higher risk of second- or third-degree AVB and AVB-related pacemaker implantation than metabolically healthy participants. However, MVPA reduced the risk of second- or third-degree AVB in the metabolically unhealthy participants, though the effect was attenuated with excessive MVPA. From this perspective, identifying and encouraging exercise in metabolically unhealthy individuals is essential. Due to its observational nature, future research should verify the preventive effects of increased MVPA on conduction block in populations with metabolic abnormalities through randomized controlled trials. Moreover, the biological mechanisms and safety of the protective effects of excessive MVPA require further verification.

## 1. Introduction 

Pacemaker implantation cases are likely to increase due to a growing aging population [1]. One of the common causes of pacemaker implantation is atrioventricular block (AVB) [2]. Permanent pacemaker implantation can cause various complications such as infection, bleeding, lead- or device-related problems, pneumothorax, cardiac tamponade, and death [3]. Additionally, the increased burden of ventricular pacing can lead to atrial fibrillation or heart failure [4]. Therefore, identifying factors that increase or decrease the AVB risk is essential.

Prior research has demonstrated that diabetes, hypertension, and obesity are positively associated with a risk of AVB [5, 6, 7]. However, there is currently a lack of information on whether a holistic approach based on metabolic status (assessed using the modified criteria for metabolic syndrome from the International Diabetes Federation [IDF], which are relatively easy to access in cardiovascular clinical settings compared to other metabolic status classifications) serves as a risk factor for AVB [8, 9]. Moreover, our previous study demonstrated that physical activity is beneficial for preventing AVB in older individuals without comorbidities [10]. However, it remains unclear whether the WHO standard recommendation of moderate-to-vigorous physical activity (MVPA) (≥150 min/week) for general well-being is beneficial in reducing the risk of AVB in metabolically healthy and metabolically unhealthy middle-aged populations [11].

Although the exact mechanism is unknown, there is some evidence that metabolic abnormalities causing chronic low-grade inflammation could be related to fibrosis of the cardiac conduction tissues [12, 13]. In this study, we hypothesized that metabolic status serves as a potential risk factor for second- or third-degree AVB and that MVPA would act as a protective factor only in metabolically unhealthy participants, considering the anti-inflammatory effects of physical activity [14]. The primary objective is to examine the relationship between metabolic status and second- or third-degree AVB in the middle-aged general population. The secondary objective is to examine the relationship between wrist-worn accelerometer-derived MVPA min/week and second- or third-degree AVB, stratified by metabolic status, and to determine whether MVPA has a protective effect on second- or third-degree AVB only in metabolically unhealthy participants. The third objective is to examine the relationship between wrist-worn accelerometer-derived MVPA min/week and high-sensitivity C-reactive protein (CRP) to support our hypothesis regarding the anti-inflammatory effects of physical activity.

## 2. Methods 

### 2.1 Study Design and Study Population 

The UK Biobank recruited over 500,000 participants aged 40 to 69 years across assessment centers in the UK between 2006 and 2010 and collected a wide range of health-related information from the participants at baseline assessment [15]. Participants’ health-related records were prospectively followed, including the International Classification of Diseases, Tenth Revision (ICD-10) and Office of Population Censuses and Surveys Classification of Interventions and Procedures-4 (OPCS4) codes. Participants’ diagnoses and health-related records were adjudicated and verified by experts, ensuring the accuracy and reliability of ICD-10 and OPCS4 codes [16].

**Fig. 1.**
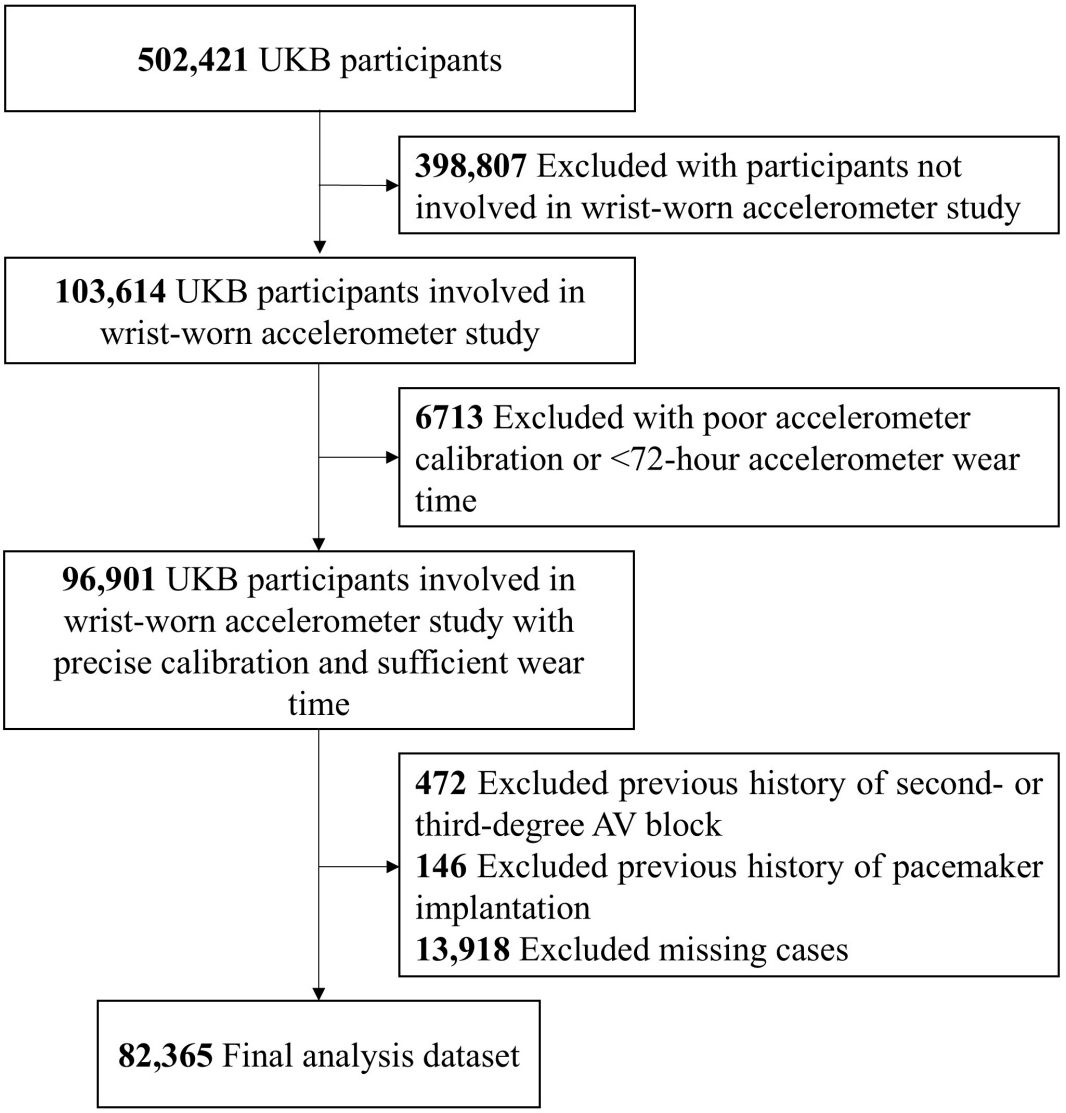
**Study population selection flowchart**. Abbreviations: AV, atrioventricular; UKB, UK Biobank.

82,365 UK Biobank participants without a history of second- or third-degree AVB or pacemaker implantation, and who were involved in accelerometer work-up, were chosen for the study population. Additionally, we excluded participants’ accelerometer data with an average acceleration under 10 mg or over 100 mg (implausible acceleration) or accelerometer wear time of less than 72 hours (poor wear-time) [17]. Fig. 1 shows the study population selection flowchart. Approval related to this study was obtained from the IRB of Yonsei University Health System (2024–3456–001), with the waiver of the need for additional informed consent. The North West Haydock Research Ethics Committee approved the UK Biobank study on June, 2021 (REC reference: 21/NM/0157), and our research was carried out under application number 77793. UK Biobank obtained informed consent from all participants and only those who did not withdraw their consent after study enrollment were included in study population.

### 2.2 Metabolic Status 

Since the UK Biobank does not have fasting-glucose data and it is difficult to accurately validate the prescriptions regarding triglycerides and high-density lipoprotein cholesterol, we used the modified criteria for metabolic syndrome from the IDF to classify the metabolic status, as used in previous studies [8, 18]. Comparison of the IDF’s criteria and modified IDF’s criteria for metabolic syndrome is shown in **Supplementary Table 1**. Metabolically unhealthy was defined as having an increased waist circumference (≥94 cm for men, ≥80 cm for women) and at least two of the following: (1) elevated serum non-fasting triglycerides (≥1.7 mmol/L), (2) low serum high-density lipoprotein (<1.03 mmol/L for men, <1.29 mmol/L for women), (3) high blood pressure (≥130 mmHg systolic or ≥85 mmHg diastolic) or a history of hypertension, (4) elevated non-fasting glucose (≥5.6 mmol/L) or a history of diabetes mellitus. Study populations who did not fulfill the criteria mentioned above were considered metabolically healthy.

For internal validation of the modified criteria for metabolic syndrome used to classify metabolic status, we compared the mean values of high-sensitivity CRP and triglyceride-to-high-density lipoprotein cholesterol ratio (a known marker of insulin resistance) between metabolically healthy and metabolically unhealthy participants. Compared to metabolically healthy participants, those with metabolically unhealthy participants had a statistically significantly higher mean high-sensitivity CRP and triglyceride-to-high-density lipoprotein cholesterol ratio (**Supplementary Fig. 1**).

### 2.3 Wrist-Worn Accelerometer Work-up to Assess MVPA 

Detailed information on the protocol and analysis methods for the wrist-worn accelerometer study at the UK Biobank can be found in a previous study [19]. 103,614 out of 502,421 UK Biobank participants involved in the wrist-worn accelerometer study. The wrist-worn accelerometer study was performed median 5.7 years after their baseline assessment. To assess MVPA, triaxial accelerometer (Axivity AX3, commercial version, Axivity Ltd, Newcastle upon Tyne, Tyne and Wear, UK) was used for up to seven days. The wrist-worn accelerometer obtained data with a time interval of 0.01 seconds and a dynamic range of ±8 g. The obtained data were adjusted to local gravity, filtered to remove the noise, and analysis was performed based on 5-second epoch. The standard deviation (SD) of all three axes <13.0 mg for more than 60 min was considered as non-wear time. We used the machine-learning model developed by Walmsley* et al*. [20] to differentiate accelerometer data as MVPA or non-MVPA. Non-wear time MVPA data was estimated based on the participants’ average daily behavior across the remaining valid days. Many previous studies have used a 7-day accelerometer workup to measure more precise and accurate physical activity, even though short-term monitoring of physical activity could introduce measurement errors due to seasonal or temporary behavioral changes [21, 22]. Although this limitation exists, a study has demonstrated that a 7-day accelerometer workup provides a reproducible measure of physical activity and was therefore used in our study to measure MVPA [23].

### 2.4 Definitions of Outcomes and Comorbidities 

The primary outcome was the occurrence of a composite of second-degree AVB or third-degree AVB. The secondary outcomes included the occurrence of each component of the primary outcome and AVB-related pacemaker implantation. These outcomes were based on ICD-10 and OPCS4 codes. Detailed definitions for the outcomes and other comorbidities are shown in **Supplementary Table 2**. We used the most recent history of comorbidities before the accelerometer work-up.

### 2.5 Derivation of Covariates 

Sociodemographic characteristics, including age, sex, white ethnicity, current smoking history, current alcohol history, Townsend deprivation index, and educational attainment, were collected using touchscreen questionnaires and computer-assisted verbal interviews. **Supplementary Table 3** provides the International Standard Classification of Education for categorizing educational attainment. Height was manually measured using a Seca 240 cm analyzer (Seca, Hamburg, Germany) and weight was measured using a Tanita BC418MA body composition analyzer (Tanita, Tokyo, Japan). Body mass index (BMI) was calculated as the weight in kg divided by the height in m^2^. Waist circumference was measured at the umbilical level using the Seca 200 cm tape measure (Seca GmbH & Co. KG, Hamburg, Germany). Blood pressure was measured using an Omron 705 IT electronic BP monitor (OMRON Healthcare, Hoofddorp, Netherlands) after 5 min of seated rest. Measurements were taken twice at 1 min intervals, and the average of the two blood pressure readings was used as a covariate. Serum glucose, lipid profiles, and high-sensitivity CRP levels were measured in the blood samples using a vacutainer after approximately 4 hours of fasting. Participants who had been taking anti-arrhythmic drugs (for example, Vaughn-Williams class 1–4), beta-blockers, or hypoglycemic agents for more than 90 days before the baseline assessment or who started such medications before the accelerometer workup for more than 90 days were considered to be taking anti-arrhythmic drugs, beta-blockers, or hypoglycemic agents for conservative sensitivity analysis. The most recent covariates before the accelerometer workup period were used in the analysis.

### 2.6 Statistical Analysis 

The baseline characteristics of metabolically healthy and metabolically unhealthy participants were summarized as mean ± SD or median (interquartile range: quartile 1–quartile 3) for continuous variables and counts and percentages for categorical variables. We used the Student’s *t*-test or Mann–Whitney U test to compare continuous variables between metabolically healthy and metabolically unhealthy participants. We used the chi-squared test or Fisher’s exact test to compare categorical variables between metabolically healthy and metabolically unhealthy participants.

The pairwise deletion method was applied to handle the missing variables. For the time-to-event analysis, follow-up started from the time when the accelerometer work-up was performed and censored at the last or loss of follow-up, or death, whichever came first. The cumulative incidence of primary and secondary outcomes was estimated by using the Kaplan-Meier method and statistically significant differences in cumulative incidence were assessed by log-rank test. Multivariable Cox regression analysis was used to estimate the adjusted hazard ratio (HR) and 95% confidence interval (CI) for the relationship between metabolic status (as a categorical variable) and the risk of primary and secondary outcomes. Potential confounders were adjusted as follows: age, sex, white ethnicity, MVPA, current smoking history, current alcohol history, and accelerometer wear time. Subgroup analysis was performed for the primary outcome to assess heterogeneity, stratified by age (<65 years vs. ≥65 years), sex, BMI (normal [<25.0 kg/m^2^] vs. overweight [25.0–29.9 kg/m^2^] vs. obese [≥30.0 kg/m^2^]), hypertension, and diabetes mellitus. The statistical significance (*p* < 0.10) of the interaction was evaluated using analysis of variance. In our multivariable models, we refrained from adjusting for potential mediators, including hypertension, diabetes, dyslipidemia, and cardiovascular diseases (for example, coronary heart disease). This decision was made to avoid introducing bias in the estimates within the causal pathway [24]. Potential multicollinearity among covariates was addressed using the variance inflation factor (VIF), and the VIF for all covariates was found to be less than 5. Proportional hazard assumptions were assessed by using Schoenfeld residual plots with Grambsch and Therneau tests. When evaluating the link between metabolic status and the incidence of AVB-related pacemaker implantation, we observed a violation of the proportional hazard assumption for metabolic status. Therefore, we stratified our analysis by time period: 0–4.0 years (period 1) and ≥4.0 years (period 2), based on the point where beta (t) significantly deviated from zero (4 years) in the Schoenfeld residual plot (**Supplementary Fig. 2**).

The relationship between MVPA min/week and the risk of primary outcome was evaluated using both categorical (decile of MVPA min/week) and continuous variables. Restricted cubic splines were used to illustrate the relationship between MVPA min/week and the HRs of the primary outcome stratified by metabolic status. The number of knots was determined using the Akaike information criterion, selecting the model with the lowest Akaike information criterion value. After analysis, three knots were chosen for plotting restricted cubic spline curves in both metabolic status category. The reference value for the spline curves was set at 0 min/week. We further categorized metabolically unhealthy participants into three groups based on the WHO’s standard (≥150 min/week) and extended (≥300 min/week) MVPA recommendation and used metabolically healthy participants as a reference to obtain adjusted HRs for the primary outcome. Finally, the correlation between MVPA min/week and high-sensitivity CRP levels in metabolically unhealthy participants was assessed by scatter plot with regression line and Spearman’s rank correlation coefficient (ρ).

Multiple sensitivity analyses were performed regarding associations between metabolic status and primary or secondary outcomes, including (1) excluding participants who had experienced the primary outcome within the first 1 or 2 years of follow-up to reduce the possibility of reverse causality; (2) additional adjustment with Vaughan Williams class 1–4 anti-arrhythmic drugs and digoxin history, which can cause AVB; (3) additional adjustment with sociodemographic factors such as Townsend deprivation index and educational attainment; and (4) excluding cases with less than 7-day accelerometer use to avoid bias from imputing MVPA during the non-wear time. Moreover, we repeated the restricted cubic spline curve analysis to illustrate the relationship between MVPA min/week and the primary outcome, (5) excluding participants with a history of beta-blocker or hypoglycemic drug use, which could affect heart rate and conduction. Statistical significance was defined as a two-tailed *p*-value of <0.05. Statistical analyses were conducted using R software version 4.4.1 (R Foundation for Statistical Computing, Vienna, Austria).

## 3. Results 

### 3.1 Study Population Baseline Characteristics 

Of 82,365 UK Biobank participants, the mean ± SD age at the start of the accelerometer workup was 62.3 ± 7.8 years and 44.1% were men. The overall median (interquartile range) MVPA min/week was 233 min/week (114–405 min/week). The distribution of counts for the overall mean acceleration and MVPA stratified by metabolic status is shown in **Supplementary Fig. 3**. The percentage of metabolically unhealthy participants who did not meet the WHO standard recommendations (MVPA ≥150 min/week) was higher than that of metabolically healthy participants (43.5% vs. 28.7%, *p* < 0.001) (**Supplementary Fig. 4**).

The baseline characteristics stratified by metabolic status are shown in Table 1. Metabolically unhealthy participants were more likely to be older and male, and had a higher BMI, larger waist circumference, higher systolic and diastolic blood pressure, and a higher prevalence of comorbidities than metabolically healthy participants (Table 1). The differences in the baseline characteristics between the non-accelerometer and accelerometer study groups in the UK Biobank registry are shown in **Supplementary Table 4**.

**Table 1.** **Baseline characteristics of metabolically healthy and unhealthy participants**.

Cohort characteristics		Total	Metabolically healthy	Metabolically unhealthy	*p* value
		(N = 82,365)	(N = 58,225)	(N = 24,140)	
Age, mean (SD), years		62.3 (7.8)	61.7 (7.9)	63.8 (7.4)	<0.001
Sex, No. (%)					<0.001
	Men	36,297 (44.1)	23,793 (40.9)	12,504 (51.8)	
	Women	46,068 (55.9)	34,432 (59.1)	11,636 (48.2)	
Race, No. (%)					0.046
	White	79,631 (96.7)	56,245 (96.6)	23,386 (96.9)	
	Others^a^	2734 (3.3)	1980 (3.4)	754 (3.1)	
Height, mean (SD), cm		169.2 (9.1)	168.8 (8.9)	170.1 (9.4)	<0.001
Weight, mean (SD), kg		76.6 (15.3)	72.1 (12.9)	87.5 (15.2)	<0.001
Body mass index, mean (SD), kg/m^2^		26.7 (4.5)	25.2 (3.6)	30.2 (4.4)	<0.001
Waist circumference, mean (SD), cm		88.3 (13.0)	83.9 (11.0)	99.1 (10.9)	<0.001
Systolic BP, mean (SD), mmHg		136.7 (18.2)	133.8 (18.1)	143.6 (16.5)	<0.001
Diastolic BP, mean (SD), mmHg		81.7 (10.0)	80.1 (9.8)	85.6 (9.5)	<0.001
Comorbidities					
	Hypertension, No. (%)	19,777 (24.0)	9986 (17.2)	9791 (40.6)	<0.001
	Diabetes mellitus, No. (%)	2708 (3.3)	562 (1.0)	2146 (8.9)	<0.001
	Dyslipidemia, No. (%)	9800 (11.9)	5054 (8.7)	4746 (19.7)	<0.001
	Coronary heart disease, No. (%)	1762 (2.1)	867 (1.5)	895 (3.7)	<0.001
	Heart failure, No. (%)	1759 (2.1)	1132 (1.9)	627 (2.6)	<0.001
	Atrial fibrillation, No. (%)	1969 (2.4)	1151 (2.0)	818 (3.4)	<0.001
Smoking history, No. (%)					<0.001
	Never or previous	76,657 (93.1)	54,423 (93.5)	22,234 (92.1)	
	Current smokers	5708 (6.9)	3802 (6.5)	1906 (7.9)	
Alcohol history, No. (%)					<0.001
	Never or Previous	4613 (5.6)	2996 (5.1)	1617 (6.7)	
	Current	77,752 (94.4)	55,229 (94.9)	22,523 (93.3)	
Townsend deprivation index, mean (SD)^b^		–1.7 (2.8)	–1.8 (2.8)	–1.6 (2.9)	<0.001
Education attainment, No. (%)^c^					<0.001
	ISCED category 1	7125 (8.7)	4294 (7.4)	2831 (11.7)	
	ISCED category 2	20,150 (24.5)	13,693 (23.5)	6457 (26.8)	
	ISCED category 3	10,828 (13.1)	7721 (13.3)	3107 (12.9)	
	ISCED category 4	4185 (5.1)	2802 (4.8)	1383 (5.7)	
	ISCED category 5	40,077 (48.6)	29,715 (51.0)	10,362 (42.9)	
Laboratory findings					
	Glucose, mean (SD), mmol/L	5.1 (1.0)	4.9 (0.7)	5.4 (1.5)	<0.001
	Triglycerides, mean (SD), mmol/L	1.7 (1.0)	1.3 (0.7)	2.4 (1.1)	<0.001
	Low-density lipoprotein, mean (SD), mmol/L	3.6 (0.8)	3.5 (0.8)	3.7 (0.9)	<0.001
	High–density lipoprotein, mean (SD), mmol/L	1.5 (0.4)	1.6 (0.4)	1.3 (0.3)	<0.001
Accelerometer data					
	Wear duration overall, median (IQR), days	6.9 (6.7–7.0)	6.9 (6.7–7.0)	6.9 (6.7–7.0)	0.563
	MVPA, median (IQR), min/week	233 (114–405)	256 (132–432)	179 (80–329)	<0.001
	Seven–day overall acceleration average, mean (SD), mg	28.2 (8.2)	29.3 (8.4)	25.5 (7.2)	<0.001
Medications					
	Vaughan–Williams class 1c, No. (%)	154 (0.2)	104 (0.2)	50 (0.2)	0.439
	Beta–blocker, No. (%)	3990 (4.8)	2079 (3.6)	1911 (7.9)	<0.001
	Vaughan–Williams class 3, No. (%)	221 (0.3)	137 (0.2)	84 (0.3)	0.006
	Non–dihydropyridine calcium–channel blocker, No. (%)	459 (0.6)	241 (0.4)	218 (0.9)	<0.001
	Digoxin, No. (%)	2805 (3.4)	1404 (2.4)	1401 (5.8)	<0.001
	Hypoglycemic drugs, No. (%)	1017 (1.2)	259 (0.4)	758 (3.1)	<0.001

Abbreviations: BP, blood pressure; IQR, interquartile range; ISCED, International Standard Classification of Education; MVPA, moderate-to-vigorous physical activity; SD, standard deviation. ^a^ Other races consist of Asian, Black, Mixed, and Others/Unknown. ^b^ Positive values of Townsend deprivation index indicate high material deprivation whereas negative values indicate relative affluence. ^c^ Educational attainment was categorized using the ISCED. A higher category indicates more years of education.

### 3.2 Metabolic Status and Primary Outcome 

**Fig. 2.**
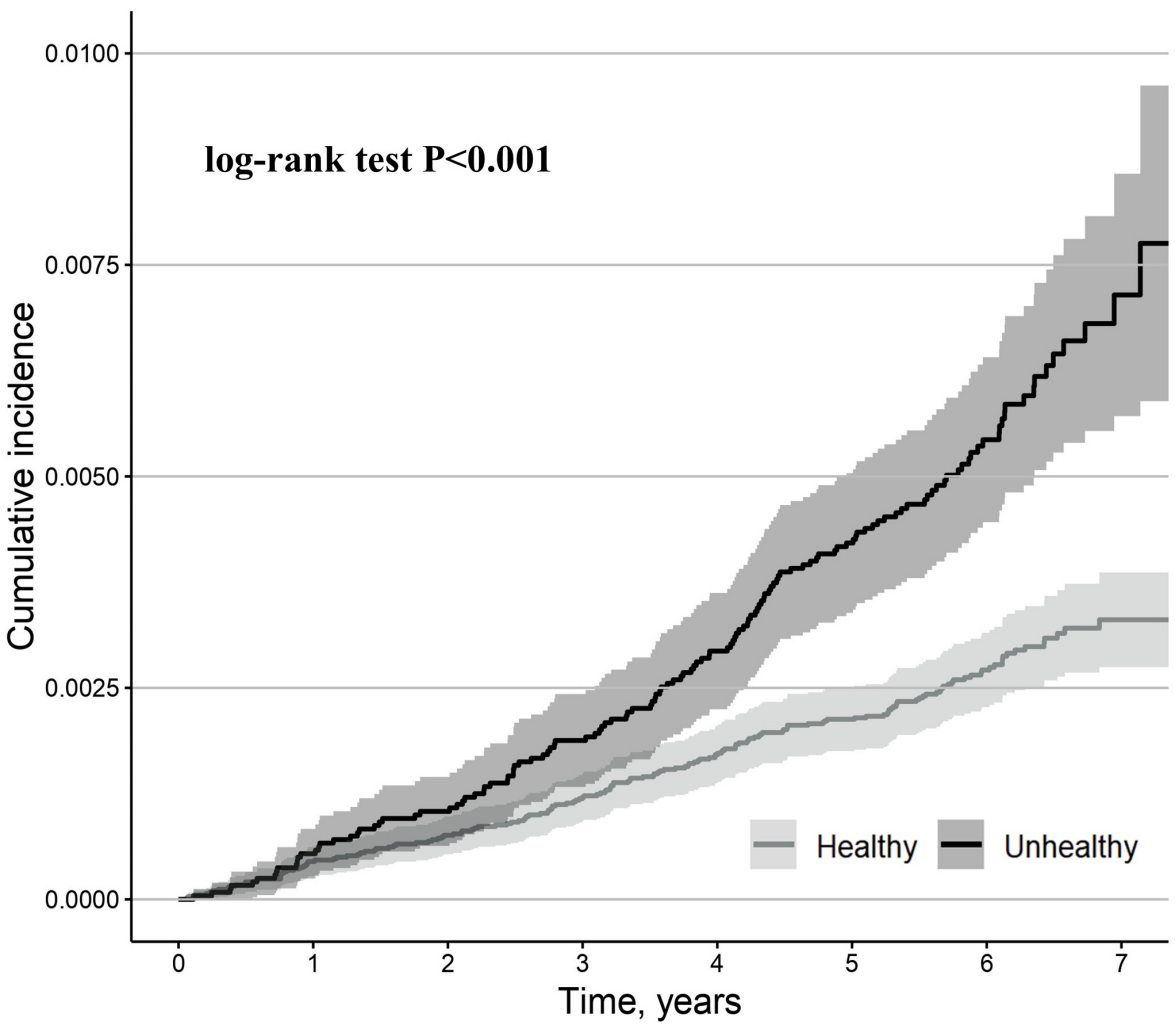
**Kaplan-Meier estimates for primary outcome (second- or third-degree AVB) stratified by metabolically healthy and metabolically unhealthy participants**. Metabolically unhealthy was defined as having an increased waist circumference (≥94 cm for men, ≥80 cm for women) and at least two of the following: (1) elevated serum non-fasting triglycerides (≥1.7 mmol/L), (2) low serum high-density lipoprotein (<1.03 mmol/L for men, <1.29 mmol/L for women), (3) high blood pressure (≥130 mmHg systolic or ≥85 mmHg diastolic) or a history of hypertension, (4) elevated non-fasting glucose (≥5.6 mmol/L) or a history of diabetes mellitus. Study populations who did not fulfill the criteria mentioned above were considered metabolically healthy. The shaded area indicates CI.

During the median (interquartile range) 6.1-year (5.6–6.6 years) follow-up, 299 cases of the primary outcome were observed. Compared to metabolically healthy participants, metabolically unhealthy participants had a higher cumulative incidence of the primary outcome (log-rank test, *p* < 0.001) (Fig. 2). Compared to metabolically healthy participants, metabolically unhealthy participants had a 58% higher incidence of the primary outcome (adjusted HR: 1.58, 95% CI: 1.25–2.00, *p* < 0.001) (Table 2). There was no significant interaction between metabolic status and age, sex, hypertension, or diabetes mellitus regarding the primary outcome (Table 3). However, subgroup analysis showed that the adjusted HRs for the normal, overweight, and obese groups were statistically significantly different (*p* for interaction = 0.053) (Table 3).

**Table 2.** **Multivariable associations between metabolic status and primary outcome (incident second- or third-degree AVB) and secondary outcomes (incident second-degree AVB, third-degree AVB, and AVB-related pacemaker implantation)**.

Metabolic status			Unadjusted HR (95% CI)	*p* value	Adjusted HR^a^ (95% CI)	*p* value
Primary outcome (second- or third-degree AVB)						
		Metabolically healthy (N = 58,225)	1.00 [Reference]	NA	1.00 [Reference]	NA
		Metabolically unhealthy (N = 24,140)	2.04 (1.62 to 2.56)	<0.001	1.58 (1.25 to 2.00)	<0.001
	N = 299 second- or third-degree AVB events; median follow-up 6.1 years (quartile 1: 5.6, quartile 3: 6.6)					
Secondary outcomes						
Second-degree AVB						
		Metabolically healthy (N = 58,225)	1.00 [Reference]	NA	1.00 [Reference]	NA
		Metabolically unhealthy (N = 24,140)	2.09 (1.47 to 2.95)	<0.001	1.59 (1.12 to 2.27)	0.010
	N = 128 second-degree AVB events; median follow-up 6.1 years (quartile 1: 5.6, quartile 3: 6.6)					
Third-degree AVB						
		Metabolically healthy (N = 58,225)	1.00 [Reference]	NA	1.00 [Reference]	NA
		Metabolically unhealthy (N = 24,140)	1.96 (1.47 to 2.63)	<0.001	1.50 (1.12 to 2.03)	0.008
	N = 184 third-degree AVB events; median follow-up 6.1 years (quartile 1: 5.6, quartile 3: 6.6)					
AVB-related pacemaker implantation						
	Follow-up duration <4 years (period 1)					
		Metabolically healthy (N = 58,225)	1.00 [Reference]	NA	1.00 [Reference]	NA
		Metabolically unhealthy (N = 24,140)	1.12 (0.73 to 1.72)	0.596	0.89 (0.58 to 1.37)	0.592
	Follow-up duration ≥4 years (period 2)					
		Metabolically healthy (N = 58,225)	1.00 [Reference]	NA	1.00 [Reference]	NA
		Metabolically unhealthy (N = 24,140)	2.95 (1.89 to 4.59)	<0.001	2.25 (1.44 to 3.52)	<0.001
	N = 177 pacemaker implantation events; median follow-up 6.1 years (quartile 1: 5.6, quartile 3: 6.6)					

Abbreviations: AVB, atrioventricular block; CI, confidence interval; HR, hazard ratio; NA, not applicable. ^a^ Model was adjusted for age, sex, white ethnicity, moderate-to-vigorous physical activity, current smoking history, current alcohol history, and accelerometer wear time.

**Table 3.** **Subgroup analysis results for the association between metabolic status and primary outcome (incident second- or third-degree AVB)**.

Subgroup		Adjusted HR^a^	*p* value	*p* value for interaction
		(95% CI)		
Age				0.449
	<65 years (N = 46,789)	1.68 (1.08 to 2.62)	0.022	
	≥65 years (N = 35,576)	1.62 (1.23 to 2.14)	<0.001	
Sex				0.188
	Men (N = 36,297)	1.78 (1.35 to 2.35)	<0.001	
	Women (N = 46,068)	1.18 (0.76 to 1.82)	0.461	
BMI				0.053
	Normal (<25.0 kg/m^2^) (N = 32,383)	0.67 (0.11 to 4.02)	0.665	
	Overweight (25.0–29.9 kg/m^2^) (N = 34,049)	1.18 (0.82 to 1.71)	0.380	
	Obese (≥30.0 kg/m^2^) (N = 15,933)	2.50 (1.39 to 4.51)	0.002	
Hypertension				0.841
	Yes (N = 19,777)	1.51 (1.05 to 2.17)	0.026	
	No (N = 62,588)	1.46 (1.06 to 2.00)	0.021	
Diabetes mellitus				0.561
	Yes (N = 2708)	2.09 (0.62 to 7.04)	0.237	
	No (N = 79,657)	1.48 (1.16 to 1.89)	0.002	

Abbreviation: BMI, body mass index. ^a^ Model was adjusted for age, sex, white ethnicity, moderate-to-vigorous physical activity, current smoking history, current alcohol history, and accelerometer wear time.

### 3.3 Metabolic Status and Secondary Outcomes 

During the median 6.1-year follow-up, 128 cases of second-degree AVB, 184 cases of third-degree AVB, and 177 cases of AVB-related pacemaker implantations were observed. 13 out of 128 cases of second-degree AVB advanced to third-degree AVB (5 out of 59 metabolically unhealthy participants [8.5%] and 8 out of 69 metabolically healthy participants [11.6%]). Most second-degree AVB, third-degree AVB, and pacemaker implantation events occurred in patients aged 70–80 years (**Supplementary Fig. 5A–C**). Most pacemaker implantations were performed within two days after the diagnosis of a second- or third-degree AVB (**Supplementary Fig. 6**).

**Fig. 3.**
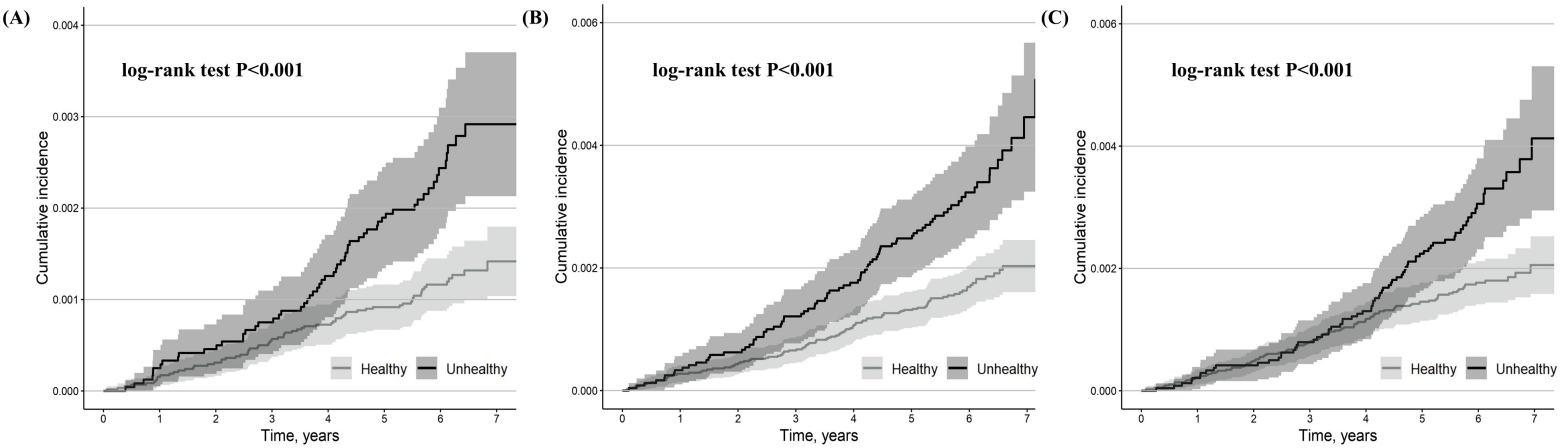
**Kaplan-Meier estimates for second-degree AVB (A), third-degree AVB (B), and second- or third-degree AVB-related pacemaker implantation (C) stratified by metabolically healthy and metabolically unhealthy participants**. The shaded area indicates CI.

Compared to metabolically healthy participants, metabolically unhealthy participants had a higher cumulative incidence of second-degree AVB, third-degree AVB, and AVB-related pacemaker implantation (log-rank test, *p* < 0.001) (Fig. 3A–C). Compared to metabolically healthy participants, metabolically unhealthy participants had a 59% higher incidence of second-degree AVB (adjusted HR: 1.59, 95% CI: 1.12–2.27, *p* = 0.010) and a 50% higher incidence of third-degree AVB (adjusted HR: 1.50, 95% CI: 1.12–2.03, *p* = 0.008) (Table 2). Compared to metabolically healthy participants, metabolically unhealthy participants had a 125% higher incidence of AVB-related pacemaker implantation (adjusted HR: 2.25, 95% CI: 1.44–3.52, *p* < 0.001) in a period of ≥4 years (period 2) (Table 2).

### 3.4 MVPA With Primary Outcome Stratified by Metabolic Status 

**Fig. 4.**
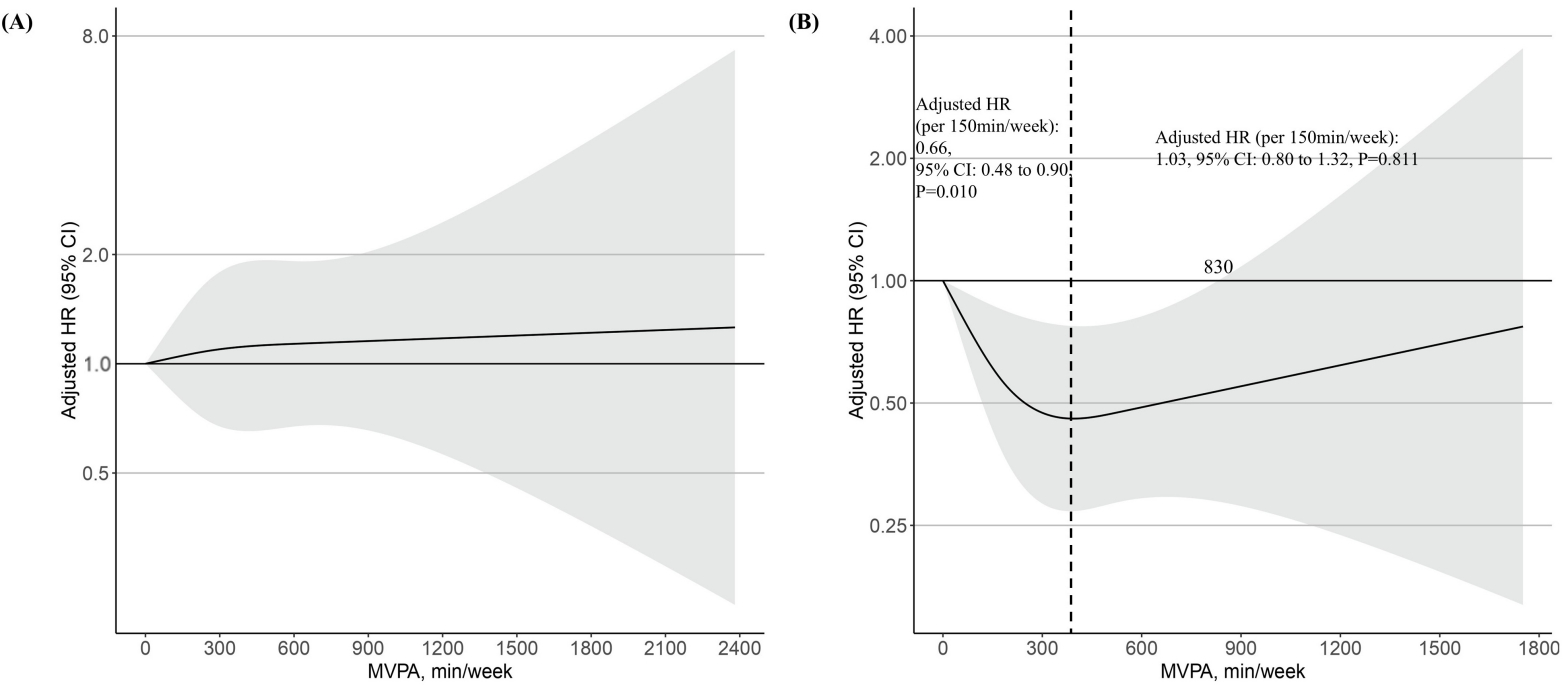
**The dose-response associations of MVPA with risk of primary outcome (incident second- or third-degree AVB) stratified by metabolically healthy (A) and metabolically unhealthy (B) participants in log scale**. The black dashed line indicates 350 min/week of MVPA, which corresponds to the lowest point for the adjusted HR. The shaded area indicates CI. Restricted cubic spline models were fitted for Cox proportional hazards model, which was adjusted for age, sex, white ethnicity, current smoking history, current alcohol history, and accelerometer wear time.

In the restricted spline curve analysis, no association was found between MVPA and risk of the primary outcome in metabolically healthy participants (Fig. 4A). In metabolically unhealthy participants, however, the risk of the primary outcome decreased with increasing MVPA until 350 min/week, but then increased, forming a J-shaped relationship (Fig. 4B). In the subgroup with less than 350 min/week of MVPA, MVPA per 150 min/week was negatively associated with a risk of the primary outcome (adjusted HR: 0.66, 95% CI: 0.48 to 0.90, *p* = 0.010) (Fig. 4B). **Supplementary Table 5** lists the relationship between the deciles of MVPA min/week and the risk of primary outcomes in metabolically unhealthy participants. Compared to decile 1 (0–27 min/week), those in decile 8 (292–379 min/week) had a 61% lower risk of primary outcome in metabolically unhealthy participants (adjusted HR: 0.39, 95% CI: 0.17–0.86, *p* = 0.020) (**Supplementary Table 5**). In the subgroup with more than 350 min/week of MVPA, MVPA per 150 min/week was positively associated with a risk of the primary outcome, though not statistically significant (adjusted HR: 1.03, 95% CI: 0.80 to 1.32, *p* = 0.811) (Fig. 4B). Excessive MVPA attenuated the beneficial effect on the primary outcome, with a threshold of 830 min/week (Fig. 4B).

Compared to metabolically healthy participants, metabolically unhealthy participants who did not meet the WHO’s standard recommendation (<150 min/week) had a 102% higher risk of the primary outcome (adjusted HR: 2.02, 95% CI: 1.53–2.67, *p* < 0.001) (Table 4). Compared to metabolically healthy participants, there was no statistically significant difference in the incidence of the primary outcome among metabolically unhealthy participants who met only the WHO’s standard recommendations (150–300 min/week) (adjusted HR: 1.28, 95% CI: 0.87–1.90, *p* = 0.209) and those who met the WHO’s extended recommendations (≥300 min/week) (adjusted HR: 1.33, 95% CI: 0.91–1.92, *p* = 0.138) (Table 4).

**Table 4.** **The joint associations of metabolic status and physical activity with incident primary outcome (second- or third-degree AVB)**.

Metabolic status and physical activity		Unadjusted HR	*p* value	Adjusted HR	*p* value
		(95% CI)		(95% CI)^a^	
Metabolically healthy (N = 58,225)		1.00 [Reference]	NA	1.00 [Reference]	NA
Metabolically unhealthy (N = 24,140)					
	Below WHO standard recommendation^b^ (N = 10,500)	2.50 (1.89 to 3.30)	<0.001	2.02 (1.53 to 2.67)	<0.001
	Meets only WHO standard recommendation^c^ (N = 6676)	1.62 (1.10 to 2.39)	0.015	1.28 (0.87 to 1.90)	0.209
	Above WHO extended recommendation^d^ (N = 6964)	1.75 (1.21 to 2.54)	0.003	1.33 (0.91 to 1.92)	0.138
MVPA per 150 min/week increase in metabolically unhealthy (<350 min/week)		0.72 (0.53 to 0.98)	0.035	0.66 (0.48 to 0.90)	0.010
MVPA per 150 min/week increase in metabolically unhealthy (≥350 min/week)		1.05 (0.83 to 1.33)	0.675	1.03 (0.80 to 1.32)	0.811

Abbreviation: WHO,World Health Organization. ^a^ Model was adjusted for age, sex, white ethnicity, current smoking history, current alcohol history, and accelerometer wear time. ^b^ Defined as <150 min/week of MVPA. ^c^ Defined as 150–300 min/week of MVPA. ^d^ Defined as ≥300 min/week of MVPA.

### 3.5 Anti-Inflammatory Effect of MVPA in Metabolically Unhealthy Participants 

In metabolically unhealthy participants, high-sensitivity CRP levels decreased as MVPA min/week increased (ρ: –0.19, *p* < 0.001) (**Supplementary Fig. 7**).

### 3.6 Sensitivity Analysis 

Study results were robust even when (1) excluding participants who had experienced the primary outcome within the first 1 or 2 years of follow-up (**Supplementary Tables 6,7**), (2) additionally adjusting for Vaughan Williams class 1–4 anti-arrhythmic drugs and digoxin history (**Supplementary Table 8**), and (3) additionally adjusting for sociodemographic factors, such as the Townsend deprivation index and educational attainment (**Supplementary Table 9**). However, when analyzing cases with complete seven-day accelerometer data, the results remained consistent, except for AVB-related pacemaker implantation, which was marginally statistically significant (adjusted HR: 1.65, 95% CI: 0.99–2.76, *p* = 0.056) (**Supplementary Table 10**). Moreover, even after excluding participants with a history of beta-blocker or hypoglycemic drug use, MVPA’s protective effect on the metabolically unhealthy population remained consistent (**Supplementary Fig. 8**).

## 4. Discussion 

In the general middle-aged population, compared to metabolically healthy participants, metabolically unhealthy participants had a higher risk of second-degree AVB, third-degree AVB, and AVB-related pacemaker implantation, and this association was stronger in obese participants. Metabolic status predicted the response to MVPA, with increased MVPA protecting second- or third-degree AVB only for those with metabolically unhealthy participants, with a threshold of 830 min/week. No statistically significant difference in the risk of second- or third-degree AVB between metabolically healthy participants and metabolically unhealthy participants who met the WHO standard recommendation (≥150 min/week) were found. Additionally, increased MVPA was negatively associated with high-sensitivity CRP levels in a metabolically unhealthy population.

### 4.1 Metabolic Status as a Risk Factor for AVB and Related Pacemaker Implantation 

Metabolically unhealthy status, defined using the criteria from the modified version of the metabolic syndrome by the IDF, was related to a higher risk of second- or third-degree AVB and AVB-related pacemaker implantation in the middle-aged general population. Prior research has demonstrated that serum glucose levels, blood pressure, and obesity are related to a higher risk of AVB [5, 6, 7, 25]. Since these factors are highly linked to a metabolically unhealthy status, and as we used the modified definition of metabolic syndrome from the IDF, our study results are plausible [12]. Although our study is observational in nature and therefore limited in its ability to assess causation, there is indirect evidence supporting a potential causal effect of metabolic indicators—such as obesity and blood pressure—demonstrated by Mendelian randomization studies. From this perspective, our results may suggest that metabolic status acts as a potential risk factor [25].

Currently, there is no definitive method for defining the metabolic status [9]. Few studies have used insulin resistance or cardiorespiratory fitness to categorize the metabolic status. However, categorizing metabolic status based on insulin resistance or cardiorespiratory fitness requires a Homeostatic Model Assessment for Insulin Resistance or a treadmill test. Approaching metabolic status using the criteria for metabolic syndrome involves assessing vital signs, clinical history, waist circumference, and laboratory findings typically obtained during follow-up in the cardiovascular outpatient department. Therefore, using these criteria to identify an unhealthy metabolic status may be more relevant in cardiovascular clinical settings to find out high risk group regarding second- or third-degree AVB and AVB-related pacemaker implantation.

### 4.2 Metabolic Status as a Predictor of Response to MVPA in AVB 

A metabolically unhealthy status, defined by a modified version of metabolic syndrome from the IDF, is often called a “low-grade chronic inflammatory status” [26]. Notably, MVPA had a protective impact on second- or third-degree AVB only in metabolically unhealthy participants who were in a relatively high inflammatory state compared with metabolically healthy participants. Inflammation is known to be associated with cardiac conduction disorders [13, 27]. Considering that MVPA has anti-inflammatory effects (also shown in our study), this finding is plausible [14]. Moreover, this finding suggests that metabolic status acts as a predictor of response to MVPA and identifies the target population for the primary prevention of second- or third-degree AVB through MVPA. Given the high percentage of metabolically unhealthy participants who do not meet the WHO’s standard recommendation (>150 min/week) (43.5%) and the finding that meeting this recommendation results in similar risk of second- or third-degree AVB compared to metabolically healthy participants, it is crucial to identify and promote MVPA for at least 150 min/week in metabolically unhealthy middle-aged individuals within clinical settings. However, the adjusted HR of second- or third-degree AVB followed a J-shaped relationship with MVPA, with a threshold of 830 min/week. Probably, excessive MVPA may lead to extreme exercise-related bradyarrhythmia which attenuates the beneficial impact of physical activity on AVB [28]. Therefore, it is advisable to encourage metabolically unhealthy participants not only to exercise at the level of the WHO’s standard recommendation (≥150 min/week), but also to avoid excessive MVPA. Accelerometers or wearable devices can help maintain a moderate level of MVPA and prevent excessive activity, thereby preserving the beneficial effects of second- or third-degree AVB.

### 4.3 Limitations 

There are several limitations in our study. First, the UK Biobank is prone to healthy volunteer selection bias, which limits the generalizability of our study results. Second, we could not assess the difference in the association between exposure and outcome by ethnicity, because most participants were Caucasian. Third, short-term monitoring of physical activity by wrist-worn accelerometer could introduce measurement errors due to seasonal or temporary behavioral changes. Fourth, since this study is observational, it is important to recognize that this study can’t completely exclude the possibility of reverse causality or residual confounders. Fifth, ICD-10 codes cannot distinguish Mobitz type 1 and 2 second-degree AVB (both types are included in the same ICD-10 code), even though Mobitz types 1 and 2 have different indications for pacemaker implantation. Specifically, pacemaker implantation is indicated in Mobitz type 1 second-degree AVB only in symptomatic patients or if the conduction delay occurs below the bundle of His, whereas pacemaker implantation is indicated in Mobitz type 2 second-degree AVB irrespective of other conditions [29]. Moreover, relying merely on physicians’ diagnosis of ICD-10 codes could potentially miss asymptomatic AVB events. Sixth, the confirmation of ICD-10 codes using electrocardiogram data was not possible because most AVB events occurred after the electrocardiogram workup period in the UK Biobank registry. Finally, this study has limitations regarding the lack of a detailed medical history of participants before the accelerometer study, which may have led to an underestimation of the prevalence of participants’ comorbidities.

## 5. Conclusion 

In the general middle-aged population, metabolically unhealthy participants had a higher risk of second- or third-degree AVB and AVB-related pacemaker implantation compared to metabolically healthy participants, and this association was stronger in obese participants. Additionally, MVPA had a protective effect on incident second- or third-degree AVB in metabolically unhealthy participants, with a threshold of 830 min/week. Given the low proportion of metabolically unhealthy participants meeting the WHO’s standard recommendation, and the observation that meeting this recommendation in metabolically unhealthy individuals results in a second- or third-degree AVB incidence comparable to that of metabolically healthy individuals, it is crucial to identify and encourage more than 150 min/week of MVPA in metabolically unhealthy individuals in clinical settings. Future research should verify the preventive effects of increased MVPA on conduction block in populations with metabolic abnormalities through randomized controlled trials. Moreover, the biological mechanisms and safety of the protective effects of excessive MVPA require further verification [28, 30].

## Availability of Data and Materials

Data supporting the findings of this study were sourced from the UK Biobank registry. However, owing to licensing restrictions, UK Biobank registry data are not publicly accessible. They can be obtained from the corresponding author upon reasonable request and with permission from the UK Biobank.

## Supplementary Material

Supplementary material associated with this article can be found, in the online version, at https://doi.org/10.31083/RCM37291.
