# Supplementary material for: Metabolic Status and Atrioventricular Block Risk: The Role of Physical Activity
Source: Rev Cardiovasc Med. 2025 May 20;26(5):37291. doi: 10.31083/RCM37291 (PMC12135636; doi:10.31083/RCM37291)
Supplement: Supplementary file 1 [file 2153-8174-26-5-37291-s1.docx]

**SUPPLEMENTARY MATERIAL**

*Metabolic Status and Atrioventricular Block Risk: The Role of Physical Activity*

| **Contents** | **Page** |
| --- | --- |
| **Supplementary Table 1.** Comparison of International Diabetes Federation’s criteria and modified International Diabetes Federation’s criteria for metabolic syndrome for Europids. | 3 |
| **Supplementary Table 2.** Definitions used for defining comorbidities. | 4 |
| **Supplementary Table 3.** ISCED definitions for categorizing educational attainment. | 5 |
| **Supplementary Table 4.** Baseline characteristic differences between the non-accelerometer study group and the accelerometer study group. | 6 |
| **Supplementary Table 5.** Association between decile of weekly MVPA as a categorical variable and incident second- or third-degree AVB in metabolically unhealthy participants. | 7 |
| **Supplementary Table 6.** Multivariable associations between metabolic status and primary outcome (incident second- or third-degree AVB) and secondary outcomes (incident second-degree AVB, third-degree AVB, and AVB-related pacemaker implantation) excluding participants with incident primary outcome in the first 1 year of follow-up. | 8 |
| **Supplementary Table 7.** Multivariable associations between metabolic status and primary outcome (incident second- or third-degree AVB) and secondary outcomes (incident second-degree AVB, third-degree AVB, and AVB-related pacemaker implantation) excluding participants with incident primary outcome in the first 2 year of follow-up. | 9 |
| **Supplementary Table 8.** Multivariable associations between metabolic status and primary outcome (incident second- or third-degree AVB) and secondary outcomes (incident second-degree AVB, third-degree AVB, and AVB-related pacemaker implantation) additionally adjusted by Vaughan Williams class 1–4 antiarrhythmic drugs and digoxin history. | 10 |
| **Supplementary Table 9.** Multivariable associations between metabolic status and primary outcome (incident second- or third-degree AVB) and secondary outcomes (incident second-degree AVB, third-degree AVB, and AVB-related pacemaker implantation) additionally adjusted by sociodemographic factors such as Townsend Deprivation Index and education attainment. | 11 |
| **Supplementary Table 10.** Multivariable associations between metabolic status and primary outcome (incident second- or third-degree AVB) and secondary outcomes (incident second-degree AVB, third-degree AVB, and AVB-related pacemaker implantation) by using cases with only complete seven-day accelerometer use. | 12 |
| **Supplementary Fig. 1.** Comparison of hsCRP (A) and TG-to-HDL-C ratio (B) between metabolically healthy and metabolically unhealthy UKB participants. | 14 |
| **Supplementary Fig. 2.** Schoenfeld residuals plot of for beta coefficient of metabolic status regarding pacemaker implantation after second- or third-degree AVB event. | 15 |
| **Supplementary Fig. 3.** Distribution of accelerometer-derived physical activity related parameters stratified by metabolically healthy and metabolically unhealthy participants. | 16 |
| **Supplementary Fig. 4.** Percentage of individuals meeting mutually exclusive WHO standard (MVPA≥150 min/week) or extended recommendation (MVPA≥300 min/week) for physical activity, stratified by metabolic status. | 17 |
| **Supplementary Fig. 5.** Distribution of age at the time of second-degree AVB event (A), age at the time of third-degree AVB event (B), and age at the time of pacemaker implantation after second- or third-degree AVB event (C) shown by histogram. | 18 |
| **Supplementary Fig. 6.** Distribution of time to pacemaker implantation after second- or third-degree AVB event, shown by histogram. | 19 |
| **Supplementary Fig. 7.** Scatter plot with regression line between MVPA min/week and log-transformed hsCRP (mg/L) in metabolically unhealthy participants. | 20 |
| **Supplementary Fig. 8.** The dose-response associations of MVPA with risk of incident second- or third-degree AVB stratified by metabolically healthy (A) and metabolically unhealthy (B) participants in log scale, excluding those with a history of beta-blocker or hypoglycemic drug use. | 21 |

**Supplementary Table 1.** Comparison of International Diabetes Federation’s criteria and modified International Diabetes Federation’s criteria for metabolic syndrome for Europids.

|  | **International Diabetes Federation** | **Modified criteria of International Diabetes Federation** |
| --- | --- | --- |
| Central obesity | ≥94 cm for men, ≥80 cm for women | ≥94 cm for men, ≥80 cm for women |
| Serum TG | ≥150 mg/dL (1.7 mmol/L) or specific treatment for this lipid abnormality | ≥ 150mg/dL (1.7 mmol/L) |
| Serum HDL-C | <40 mg/dL (1.03 mmol/L) for men, <50 mg/dL (1.29 mmol/L) for women or specific treatment for this lipid abnormality | <40 mg/dL (1.03 mmol/L) for men, <50 mg/dL (1.29 mmol/L) for women |
| Blood pressure | Systolic BP ≥130 mmHg or Diastolic BP ≥85 mmHg diastolic or treatment of previously diagnosed hypertension | Systolic BP ≥130 mmHg or Diastolic BP ≥85 mmHg diastolic or a history of hypertension |
| Plasma glucose | Fasting glucose ≥ 100 mg/dL (5.6 mmol/L) or previously diagnosed type 2 diabetes | Non-fasting glucose ≥100 mg/dL (5.6 mmol/L) or a history of diabetes mellitus |

To define metabolic syndrome based on International Diabetes Federation’s criteria and modified International Diabetes Federation’s criteria, they must have three or more factors including central obesity.

Abbreviation: HDL-C, high-density lipoprotein cholesterol; TG, triglyceride.

**Supplementary Table 2.** Definitions used for defining comorbidities.

| **Comorbidities** | **UK Biobank** | |
| --- | --- | --- |
|  | **Definitions** | **Used codes or conditions** |
| Atrial fibrillation | Defined from UK Biobank self-report or diagnosis^a^ | Self-reported non-cancer illness code: 1471, 1483  *ICD-10*: I48 |
| Coronary heart disease | Defined from UK Biobank self-report or diagnosis^a^ | Self-reported non-cancer illness code: 1075  *ICD-10*: I25.2, I25.6, I25.8, I25.9 |
| Diabetes mellitus | Defined from UK Biobank self-report or diagnosis^a^ | Self-reported non-cancer illness code: 1220, 1222, 1223, 1521  *ICD-10*: E10, E11, E12, E13, E14 |
| Dyslipidemia | Defined from UK Biobank self-report or diagnosis^a^ | Self-reported non-cancer illness code: 1473  *ICD-10*: E78 |
| Heart failure | Defined from UK Biobank self-report or diagnosis^a^ | Self-reported non-cancer illness code: 1076  *ICD-10*: I11.0, I50, I97.1 |
| Hypertension | Defined from UK Biobank self-report or diagnosis^a^ | Self-reported non-cancer illness code: 1065, 1072  *ICD-10*: I10, I11, I12, I13, I15 |
| AVB-related Pacemaker implantation | Defined from UK Biobank self-report or diagnosis^a^ | OPCS4: K605, K606, K615, and K616, following ICD-10: I44.1, I44.2, I44.3, I45.8, and I45.9 |
| Second- or third-degree AVB | Defined from UK Biobank self-report or diagnosis^a^ | *ICD-10*: I44.1, I44.2, I44.3, I45.8, I45.9 |
| Second-degree AVB | Defined from UK Biobank self-report or diagnosis^a^ | *ICD-10*: I44.1, I44.3 |
| Third-degree AVB | Defined from UK Biobank self-report or diagnosis^a^ | *ICD-10*: I44.2, I45.8, I45.9 |

Abbreviation: AVB, atrioventricular block; *ICD-10*, International Classification of Diseases Tenth Revision; *OPCS4*, OPCS Classification of Interventions and Procedures Version 4.

^a^ To ensure accuracy, comorbidities were established based on more than one hospital-inpatient or two outpatients (=primary care in United Kingdom) records of ICD-10 codes in the database.

**Supplementary Table 3.** ISCED definitions for categorizing educational attainment.

| **Qualification (As reported in UK Biobank through touchscreen question)** | **ISCED** | **Years of education** |
| --- | --- | --- |
| College or university degree | 5 | 20 |
| A levels/AS levels or equivalent | 3 | 13 |
| O levels/GCSEs or equivalent | 2 | 10 |
| CSEs or equivalent | 2 | 10 |
| NVQ or HND or HNC or equivalent | 5 | 19 |
| Other professional qualification such as nursing or teaching | 4 | 15 |
| None of the above | 1 | 7 |

Abbreviations: ISCED, International Standard Classification of Education.

**Supplementary Table 4.** Baseline characteristic differences between the non-accelerometer study group and the accelerometer study group.

| **Cohort characteristics** | **Non-accelerometer study group (N=398,807)** | **Accelerometer study group (N=103,614)** | **P value** |
| --- | --- | --- | --- |
| Age, mean (SD), years | 57.2 (8.2) | 56.6 (7.8) | <0.001 |
| Sex, No. (%) |  |  | <0.001 |
| Men | 183,721 (46.1) | 45,368 (43.8) |  |
| Women | 215,086 (53.9) | 58,246 (56.2) |  |
| Race, No. (%) |  |  | <0.001 |
| White | 372,688 (93.7) | 99,936 (96.5) |  |
| Others^a^ | 25,269 (6.3) | 3630 (3.5) |  |
| Height, mean (SD), cm | 168.3 (9.3) | 169.1 (9.1) | <0.001 |
| Weight, mean (SD), kg | 78.4 (16.1) | 76.7 (15.4) | <0.001 |
| Body mass index, mean (SD), kg/m^2^ | 27.6 (4.9) | 26.7 (4.5) | <0.001 |
| Waist circumference, mean (SD), cm | 90.8 (13.5) | 88.4 (13.1) | <0.001 |
| Systolic BP, mean (SD), mmHg | 138.4 (18.9) | 136.5 (18.3) | <0.001 |
| Diastolic BP, mean (SD), mmHg | 82.4 (10.2) | 81.6 (10.1) | <0.001 |
| Comorbidities |  |  |  |
| Hypertension, No. (%) | 120,283 (30.2) | 24,974 (24.1) | <0.001 |
| Diabetes mellitus, No. (%) | 23,039 (5.8) | 3540 (3.4) | <0.001 |
| Dyslipidemia, No. (%) | 60,877 (15.3) | 12,426 (12.0) | <0.001 |
| Smoking history, No. (%) |  |  | <0.001 |
| Never or previous | 352,269 (88.5) | 96,298 (93.0) |  |
| Current smokers | 45,694 (11.5) | 7269 (7.0) |  |
| Alcohol history, No. (%) |  |  | <0.001 |
| Never or Previous | 36,177 (8.9) | 5952 (5.7) |  |
| Current | 362,630 (91.1) | 97,662 (94.3) |  |
| Townsend deprivation index, mean (SD)^b^ | -1.2 (3.2) | -1.7 (2.8) | <0.001 |

Abbreviations: BP, blood pressure; IQR, interquartile range; SD, standard deviation.

^a^ Other races consist of Asian, Black, Mixed, and Others/Unknown.

^b^ Positive values of Townsend deprivation index indicate high material deprivation whereas negative values indicate relative affluence.

**Supplementary Table 5.** Association between decile of weekly MVPA as a categorical variable and incident second- or third-degree AVB in metabolically unhealthy participants.

| **Incident second- or third-degree AVB** | | | | | |
| --- | --- | --- | --- | --- | --- |
| *Adjusted for age, sex, white ethnicity, current smoking history, current alcohol history, and accelerometer wear time.* | | | | | |
| MVPA decile | MVPA min/week | HR (95% CI) | P value | HR (95% CI) | P value |
| 1 | 0–27 | 1.00 [Reference] | NA | 2.60 (1.17 to 5.78) | 0.020 |
| 2 | 28–61 | 0.81 (0.42 to 1.56) | 0.532 | 2.11 (0.94 to 4.74) | 0.072 |
| 3 | 62–97 | 0.64 (0.32 to 1.28) | 0.208 | 1.66 (0.72 to 3.85) | 0.235 |
| 4 | 98–135 | 0.79 (0.41 to 1.52) | 0.473 | 2.04 (0.91 to 4.58) | 0.085 |
| 5 | 136–178 | 0.59 (0.29 to 1.19) | 0.139 | 1.52 (0.65 to 3.56) | 0.335 |
| 6 | 179–228 | 0.49 (0.23 to 1.03) | 0.060 | 1.27 (0.52 to 3.06) | 0.600 |
| 7 | 229–291 | 0.39 (0.17 to 0.85) | 0.019 | 0.99 (0.40 to 2.51) | 0.991 |
| 8 | 292–379 | 0.39 (0.17 to 0.86) | 0.020 | 1.00 [Reference] | NA |
| 9 | 380–527 | 0.41 (0.19 to 0.89) | 0.025 | 1.07 (0.43 to 2.63) | 0.886 |
| 10 | ≥528 | 0.64 (0.33 to 1.26) | 0.198 | 1.67 (0.74 to 3.75) | 0.214 |
| N=136 second- or third-degree AVB events; median follow-up 6.1 years (quartile 1: 5.6, quartile 3: 6.6) | | | | | |

Abbreviations: AVB, atrioventricular block; CI, confidence interval; HR, hazard ratio; MVPA, moderate-to-vigorous physical activity; NA, not applicable.

**Supplementary Table 6.** Multivariable associations between metabolic status and primary outcome (incident second- or third-degree AVB) and secondary outcomes (incident second-degree AVB, third-degree AVB, and AVB-related pacemaker implantation) excluding participants with incident primary outcome in the first 1 year of follow-up.

| **Metabolic status** | **Unadjusted HR (95% CI)** | **P value** | **Adjusted HR**^a^ **(95% CI)** | **P value** |
| --- | --- | --- | --- | --- |
| **Primary outcome (second- or third-degree AVB)** | | | | |
| Metabolically healthy (N=58,199) | 1.00 [Reference] | NA | 1.00 [Reference] | NA |
| Metabolically unhealthy (N=24,127) | 2.20 (1.72 to 2.80) | <0.001 | 1.72 (1.34 to 2.20) | <0.001 |
| **Secondary outcomes** | | | | |
| **Second-degree AVB** | | | | |
| Metabolically healthy (N=58,199) | 1.00 [Reference] | NA | 1.00 [Reference] | NA |
| Metabolically unhealthy (N=24,127) | 2.20 (1.52 to 3.18) | <0.001 | 1.70 (1.17 to 2.49) | 0.006 |
| **Third-degree AVB** | | | | |
| Metabolically healthy (N=58,199) | 1.00 [Reference] | NA | 1.00 [Reference] | NA |
| Metabolically unhealthy (N=24,127) | 2.13 (1.56 to 2.91) | <0.001 | 1.65 (1.20 to 2.26) | 0.002 |
| **AVB-related pacemaker implantation** | | | | |
| *Follow-up duration* $<$*4 years (period 1)* |  |  |  |  |
| Metabolically healthy (N=58,199) | 1.00 [Reference] | NA | 1.00 [Reference] | NA |
| Metabolically unhealthy (N=24,127) | 1.24 (0.77 to 1.99) | 0.376 | 0.96 (0.59 to 1.54) | 0.855 |
| *Follow-up duration* $\geq$*4 years (period 2)* |  |  |  |  |
| Metabolically healthy (N=58,199) | 1.00 [Reference] | NA | 1.00 [Reference] | NA |
| Metabolically unhealthy (N=24,127) | 2.95 (1.89 to 4.59) | <0.001 | 2.28 (1.46 to 3.56) | <0.001 |

Abbreviations: AVB, atrioventricular block; CI, confidence interval; HR, hazard ratio; NA, not applicable.

^a^ Model was adjusted for age, sex, white ethnicity, moderate-to-vigorous physical activity, current smoking history, current alcohol history, and accelerometer wear time.

**Supplementary Table 7.** Multivariable associations between metabolic status and primary outcome (incident second- or third-degree AVB) and secondary outcomes (incident second-degree AVB, third-degree AVB, and AVB-related pacemaker implantation) excluding participants with incident primary outcome in the first 2 year of follow-up.

| **Metabolic status** | **Unadjusted HR (95% CI)** | **P value** | **Adjusted HR**^a^ **(95% CI)** | **P value** |
| --- | --- | --- | --- | --- |
| **Primary outcome (second- or third-degree AVB)** | | | | |
| Metabolically healthy (N=58,181) | 1.00 [Reference] | NA | 1.00 [Reference] | NA |
| Metabolically unhealthy (N=24,115) | 2.29 (1.77 to 2.96) | <0.001 | 1.78 (1.37 to 2.32) | <0.001 |
| **Secondary outcomes** | | | | |
| **Second-degree AVB** | | | | |
| Metabolically healthy (N=58,181) | 1.00 [Reference] | NA | 1.00 [Reference] | NA |
| Metabolically unhealthy (N=24,115) | 2.35 (1.58 to 3.49) | <0.001 | 1.79 (1.20 to 2.69) | 0.005 |
| **Third-degree AVB** | | | | |
| Metabolically healthy (N=58,181) | 1.00 [Reference] | NA | 1.00 [Reference] | NA |
| Metabolically unhealthy (N=24,115) | 2.19 (1.58 to 3.05) | <0.001 | 1.70 (1.21 to 2.38) | 0.002 |
| **AVB-related pacemaker implantation^b^** | | | | |
| Metabolically healthy (N=58,181) | 1.00 [Reference] | NA | 1.00 [Reference] | NA |
| Metabolically unhealthy (N=24,115) | 2.15 (1.54 to 3.01) | <0.001 | 1.65 (1.17 to 2.32) | 0.004 |

Abbreviations: AVB, atrioventricular block; CI, confidence interval; HR, hazard ratio; NA, not applicable.

^a^ Model was adjusted for age, sex, white ethnicity, moderate-to-vigorous physical activity, current smoking history, current alcohol history, and accelerometer wear time.

^b^ Analysis was not stratified by time period due to the Grambsch and Therneau test results not being statistically significant after excluding participants with incident primary outcomes in the first 2 years of follow-up.

**Supplementary Table 8.** Multivariable associations between metabolic status and primary outcome (incident second- or third-degree AVB) and secondary outcomes (incident second-degree AVB, third-degree AVB, and AVB-related pacemaker implantation) additionally adjusted by Vaughan Williams class 1–4 antiarrhythmic drugs and digoxin history.

| **Metabolic status** | **Unadjusted HR (95% CI)** | **P value** | **Adjusted HR**^a^ **(95% CI)** | **P value** |
| --- | --- | --- | --- | --- |
| **Primary outcome (second- or third-degree AVB)** | | | | |
| Metabolically healthy (N=58,225) | 1.00 [Reference] | NA | 1.00 [Reference] | NA |
| Metabolically unhealthy (N=24,140) | 2.04 (1.62 to 2.56) | <0.001 | 1.59 (1.26 to 2.00) | <0.001 |
| **Secondary outcomes** | | | | |
| **Second-degree AVB** | | | | |
| Metabolically healthy (N=58,225) | 1.00 [Reference] | NA | 1.00 [Reference] | NA |
| Metabolically unhealthy (N=24,140) | 2.09 (1.47 to 2.95) | <0.001 | 1.58 (1.11 to 2.26) | 0.012 |
| **Third-degree AVB** | | | | |
| Metabolically healthy (N=58,225) | 1.00 [Reference] | NA | 1.00 [Reference] | NA |
| Metabolically unhealthy (N=24,140) | 1.96 (1.47 to 2.63) | <0.001 | 1.51 (1.12 to 2.04) | 0.007 |
| **AVB-related pacemaker implantation** | | | | |
| *Follow-up duration* $<$*4 years (period 1)* |  |  |  |  |
| Metabolically healthy (N=58,225) | 1.00 [Reference] | NA | 1.00 [Reference] | NA |
| Metabolically unhealthy (N=24,140) | 1.12 (0.73 to 1.72) | 0.596 | 0.91 (0.59 to 1.39) | 0.649 |
| *Follow-up duration* $\geq$*4 years (period 2)* |  |  |  |  |
| Metabolically healthy (N=58,225) | 1.00 [Reference] | NA | 1.00 [Reference] | NA |
| Metabolically unhealthy (N=24,140) | 2.95 (1.89 to 4.59) | <0.001 | 2.30 (1.47 to 3.60) | <0.001 |

Abbreviations: AVB, atrioventricular block; CI, confidence interval; HR, hazard ratio; NA, not applicable.

^a^ Model was adjusted for age, sex, white ethnicity, moderate-to-vigorous physical activity, current smoking history, current alcohol history, accelerometer wear time, and additional adjustment with Vaughan Williams class 1–4 antiarrhythmic drugs and digoxin history.

**Supplementary Table 9.** Multivariable associations between metabolic status and primary outcome (incident second- or third-degree AVB) and secondary outcomes (incident second-degree AVB, third-degree AVB, and AVB-related pacemaker implantation) additionally adjusted by sociodemographic factors such as Townsend Deprivation Index and education attainment.

| **Metabolic status** | **Unadjusted HR (95% CI)** | **P value** | **Adjusted HR**^a^ **(95% CI)** | **P value** |
| --- | --- | --- | --- | --- |
| **Primary outcome (second- or third-degree AVB)** | | | | |
| Metabolically healthy (N=58,225) | 1.00 [Reference] | NA | 1.00 [Reference] | NA |
| Metabolically unhealthy (N=24,140) | 2.04 (1.62 to 2.56) | <0.001 | 1.60 (1.26 to 2.02) | <0.001 |
| **Secondary outcomes** | | | | |
| **Second-degree AVB** | | | | |
| Metabolically healthy (N=58,225) | 1.00 [Reference] | NA | 1.00 [Reference] | NA |
| Metabolically unhealthy (N=24,140) | 2.09 (1.47 to 2.95) | <0.001 | 1.60 (1.12 to 2.29) | 0.010 |
| **Third-degree AVB** | | | | |
| Metabolically healthy (N=58,225) | 1.00 [Reference] | NA | 1.00 [Reference] | NA |
| Metabolically unhealthy (N=24,140) | 1.96 (1.47 to 2.63) | <0.001 | 1.50 (1.11 to 2.02) | 0.008 |
| **AVB-related pacemaker implantation** | | | | |
| *Follow-up duration* $<$*4 years (period 1)* |  |  |  |  |
| Metabolically healthy (N=58,225) | 1.00 [Reference] | NA | 1.00 [Reference] | NA |
| Metabolically unhealthy (N=24,140) | 1.12 (0.73 to 1.72) | 0.596 | 0.89 (0.58 to 1.37) | 0.600 |
| *Follow-up duration* $\geq$*4 years (period 2)* |  |  |  |  |
| Metabolically healthy (N=58,225) | 1.00 [Reference] | NA | 1.00 [Reference] | NA |
| Metabolically unhealthy (N=24,140) | 2.95 (1.89 to 4.59) | <0.001 | 2.28 (1.45 to 3.57) | <0.001 |

Abbreviations: AVB, atrioventricular block; CI, confidence interval; HR, hazard ratio; NA, not applicable.

^a^ Model was adjusted for age, sex, white ethnicity, moderate-to-vigorous physical activity, current smoking history, current alcohol history, accelerometer wear time, and additional adjustment with Townsend Deprivation Index and education attainment.

**Supplementary Table 10.** Multivariable associations between metabolic status and primary outcome (incident second- or third-degree AVB) and secondary outcomes (incident second-degree AVB, third-degree AVB, and AVB-related pacemaker implantation) by using cases with only complete seven-day accelerometer use.

| **Metabolic status** | **Unadjusted HR (95% CI)** | **P value** | **Adjusted HR**^a^ **(95% CI)** | **P value** |
| --- | --- | --- | --- | --- |
| **Primary outcome (second- or third-degree AVB)** | | | | |
| Metabolically healthy (N=17,820) | 1.00 [Reference] | NA | 1.00 [Reference] | NA |
| Metabolically unhealthy (N=7480) | 2.24 (1.52 to 3.30) | <0.001 | 1.80 (1.21 to 2.68) | 0.004 |
| **Secondary outcomes** | | | | |
| **Second-degree AVB** | | | | |
| Metabolically healthy (N=17,820) | 1.00 [Reference] | NA | 1.00 [Reference] | NA |
| Metabolically unhealthy (N=7480) | 2.66 (1.45 to 4.87) | 0.002 | 1.91 (1.03 to 3.56) | 0.040 |
| **Third-degree AVB** | | | | |
| Metabolically healthy (N=17,820) | 1.00 [Reference] | NA | 1.00 [Reference] | NA |
| Metabolically unhealthy (N=7480) | 2.07 (1.26 to 3.40) | 0.004 | 1.71 (1.03 to 2.84) | 0.037 |
| **AVB-related pacemaker implantation^b^** | | | | |
| Metabolically healthy (N=17,820) | 1.00 [Reference] | NA | 1.00 [Reference] | NA |
| Metabolically unhealthy (N=7480) | 2.06 (1.24 to 3.40) | 0.005 | 1.65 (0.99 to 2.76) | 0.056 |

Abbreviations: AVB, atrioventricular block; CI, confidence interval; HR, hazard ratio; NA, not applicable.

^a^ Model was adjusted for age, sex, white ethnicity, moderate-to-vigorous physical activity, current smoking history, and current alcohol history.

^b^ Analysis was not stratified by time period due to the Grambsch and Therneau test results not being statistically significant after using cases with only complete seven-day accelerometer use.

**Supplementary Fig. 1.** Comparison of hsCRP (A) and TG-to-HDL-C ratio (B) between metabolically healthy and metabolically unhealthy UKB participants.


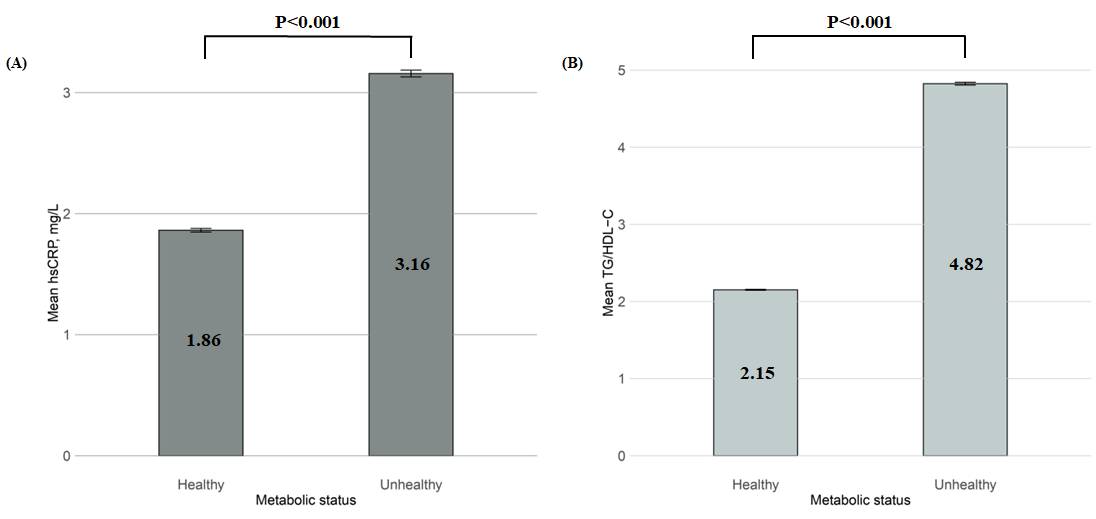


Error bars are shown as black solid lines at the top of the bar plot and mean values in each category are shown in the middle of the bar plot.

The TG-to-HDL-C ratio was calculated using the unit of mg/dL for both TG and HDL-C.

Abbreviation: HDL-C, high-density lipoprotein cholesterol; hsCRP, high-sensitivity C-reactive protein; TG, triglyceride; UKB, UK Biobank.

**Supplementary Fig. 2.** Schoenfeld residuals plot of for beta coefficient of metabolic status regarding pacemaker implantation after second- or third-degree AVB event.


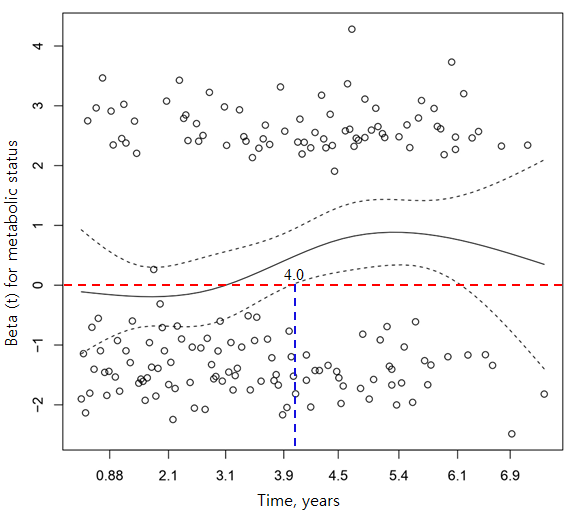


The blue dashed line indicates the time when beta (t) significantly deviates from zero (4.0 years).

The red dashed line represents the horizontal line where beta (t) equals to zero.

Cox proportional hazard model was adjusted for age, sex, white ethnicity, current smoking history, current alcohol history, moderate-to-vigorous physical activity, and accelerometer wear time.

Abbreviation: AVB, atrioventricular block.

**Supplementary Fig. 3.** Distribution of accelerometer-derived physical activity related parameters stratified by metabolically healthy and metabolically unhealthy participants.


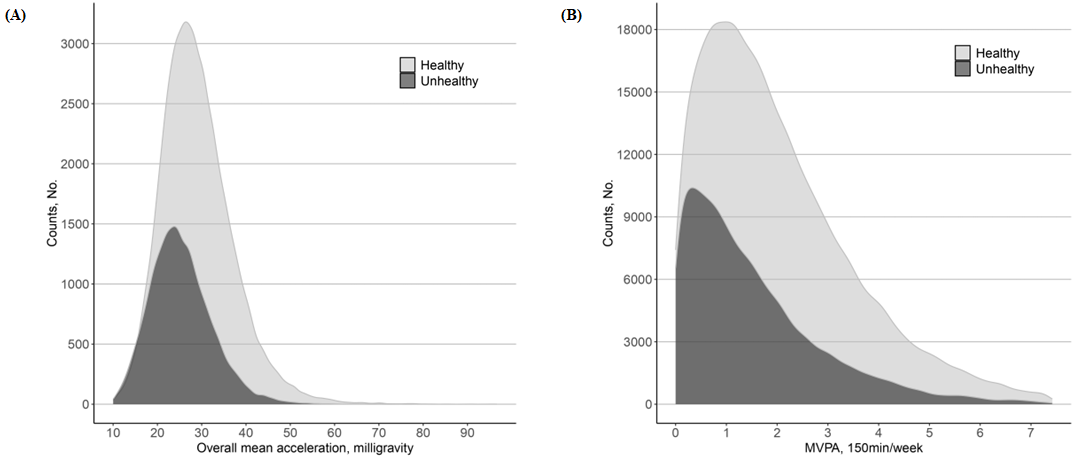


Panel (A) depicts overall mean acceleration and panel (B) depicts MVPA 150min/week.

Upper 1% percentile was removed for graphical purpose.

Abbreviation: MVPA, moderate-to-vigorous physical activity.

**Supplementary Fig. 4.** Percentage of individuals meeting mutually exclusive WHO standard (MVPA≥150 min/week) or extended recommendation (MVPA≥300 min/week) for physical activity, stratified by metabolic status.


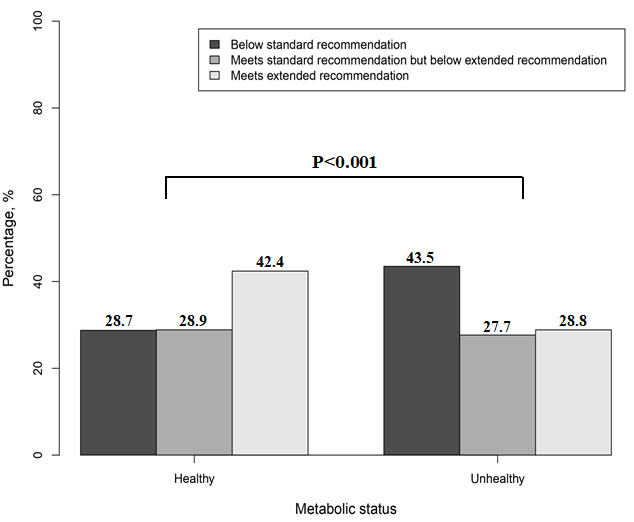


Abbreviation: MVPA, moderate-to-vigorous physical activity; WHO World Health Organization.

**Supplementary Fig. 5.** Distribution of age at the time of second-degree AVB event (A), age at the time of third-degree AVB event (B), and age at the time of pacemaker implantation after second- or third-degree AVB event (C) shown by histogram.


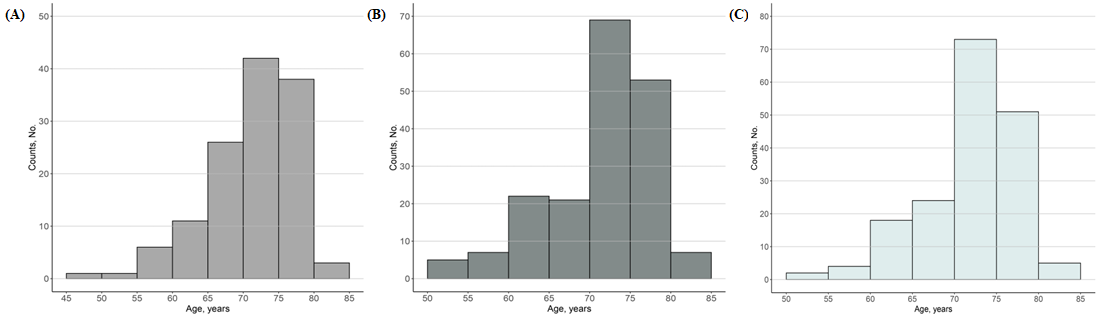


Abbreviation: AVB, atrioventricular block.

**Supplementary Fig. 6.** Distribution of time to pacemaker implantation after second- or third-degree AVB event, shown by histogram.


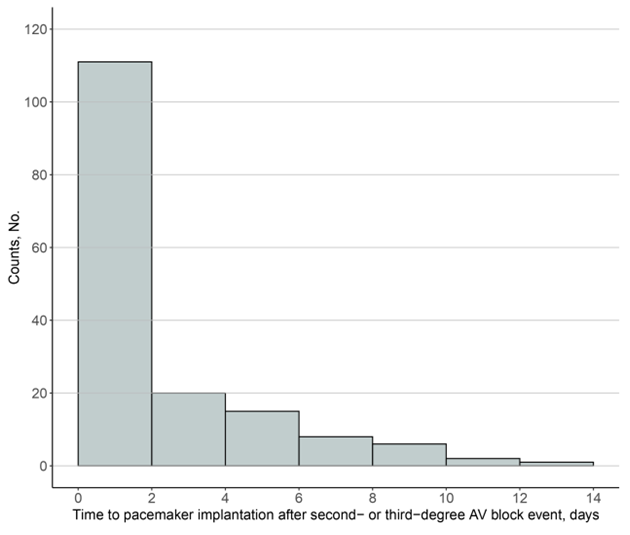


Abbreviation: AVB, atrioventricular block.

**Supplementary Fig. 7.** Scatter plot with regression line between MVPA min/week and log-transformed hsCRP (mg/L) in metabolically unhealthy participants.


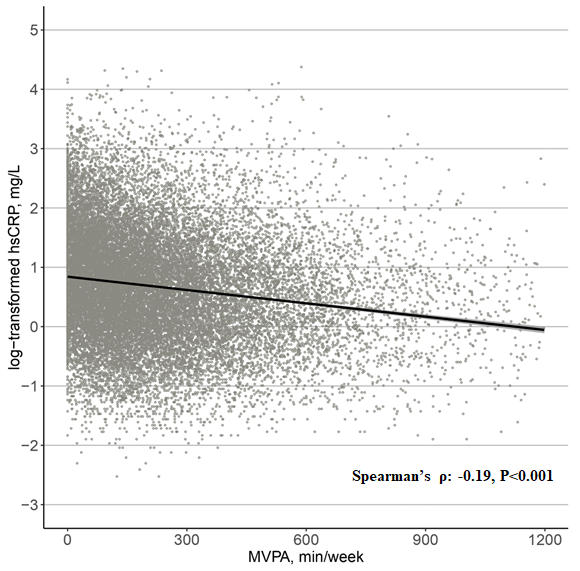


Abbreviations: CI, confidence interval; hsCRP, high-sensitivity C-reactive protein; MVPA, moderate-to-vigorous physical activity; UKB, UK Biobank.

The solid line indicates the regression line, and the shaded area indicates 95% CI.

**Supplementary Fig. 8.** The dose-response associations of MVPA with risk of incident second- or third-degree AVB stratified by metabolically healthy (A) and metabolically unhealthy (B) participants in log scale, excluding those with a history of beta-blocker or hypoglycemic drug use.


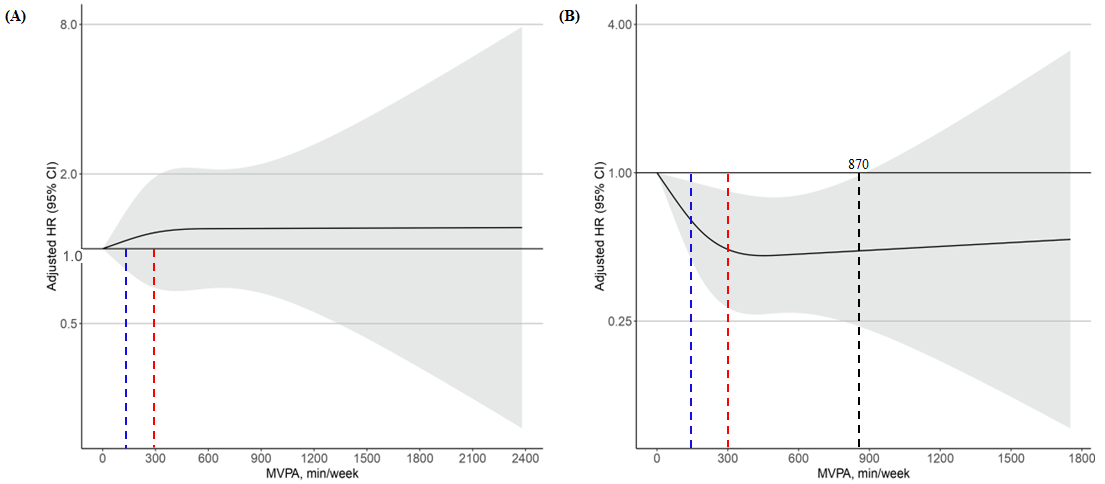


The metabolic status was classified using modified criteria for metabolic syndrome established by the International Diabetes Federation. Specifically, metabolically unhealthy was defined as having an increased waist circumference (≥94 cm for men, ≥80 cm for women) and at least two of the following: (1) elevated serum non-fasting triglycerides (≥1.7 mmol/L), (2) low serum high-density lipoprotein (<1.03 mmol/L for men, <1.29 mmol/L for women), (3) high blood pressure (≥130 mmHg systolic or ≥85 mmHg diastolic) or a history of hypertension, (4) elevated non-fasting glucose (≥5.6 mmol/L) or a history of diabetes mellitus. Participants who were not classified as metabolically unhealthy were considered metabolically healthy.

The blue dashed line indicates the minimum MVPA value according to the WHO standard recommendation ($\geq$150 min/week), while the red dashed line indicates the minimum MVPA value according to the WHO extended recommendation ($\geq$300 min/week). The black dashed line indicates the MVPA value of the x-intercept of HRs upper 95% CI (threshold).

Restricted cubic spline models were fitted for Cox proportional hazard model, which was adjusted for age, sex, white ethnicity, current smoking history, current alcohol history, and accelerometer wear time.

Abbreviation: AVB, atrioventricular block; CI, confidence interval; HR, hazard ratio; MVPA, moderate-to-vigorous physical activity.
